# Loci and candidate genes conferring resistance to soybean cyst nematode HG type 2.5.7

**DOI:** 10.1186/s12864-017-3843-y

**Published:** 2017-06-14

**Authors:** Xue Zhao, Weili Teng, Yinghui Li, Dongyuan Liu, Guanglu Cao, Dongmei Li, Lijuan Qiu, Hongkun Zheng, Yingpeng Han, Wenbin Li

**Affiliations:** 1 Key Laboratory of Soybean Biology in Chinese Ministry of Education (Key Laboratory of Soybean Biology and Breeding/Genetics of Chinese Agriculture Ministry), Northeast Agricultural University, Harbin, 150030 China; 2 Institute of Crop Science, National Key Facility for Crop Gene Resources and Genetic Improvement (NFCRI), Chinese Academy of Agricultural Sciences, Beijing, 100081 China; 3 Bioinformatics Division, Biomarker Technologies Corporation, Beijing, 101300 China

**Keywords:** Genome-wide association mapping, Soybean cyst nematode resistance, HG type 2.5.7, Candidate genes

## Abstract

**Background:**

Soybean (*Glycine max* L. Merr.) cyst nematode (SCN, *Heterodera glycines* I,) is a major pest of soybean worldwide. The most effective strategy to control this pest involves the use of resistant cultivars. The aim of the present study was to investigate the genome-wide genetic architecture of resistance to SCN HG Type 2.5.7 (race 1) in landrace and elite cultivated soybeans.

**Results:**

A total of 200 diverse soybean accessions were screened for resistance to SCN HG Type 2.5.7 and genotyped through sequencing using the Specific Locus Amplified Fragment Sequencing (SLAF-seq) approach with a 6.14-fold average sequencing depth. A total of 33,194 SNPs were identified with minor allele frequencies (MAF) over 4%, covering 97% of all the genotypes. Genome-wide association mapping (GWAS) revealed thirteen SNPs associated with resistance to SCN HG Type 2.5.7. These SNPs were distributed on five chromosomes (Chr), including Chr7, 8, 14, 15 and 18. Four SNPs were novel resistance loci and nine SNPs were located near known QTL. A total of 30 genes were identified as candidate genes underlying SCN resistance.

**Conclusions:**

A total of sixteen novel soybean accessions were identified with significant resistance to HG Type 2.5.7. The beneficial alleles and candidate genes identified by GWAS might be valuable for improving marker-assisted breeding efficiency and exploring the molecular mechanisms underlying SCN resistance.

**Electronic supplementary material:**

The online version of this article (doi:10.1186/s12864-017-3843-y) contains supplementary material, which is available to authorized users.

## Background

Soybean cyst nematode (SCN, *Heterodera glycines* Ichinohe) is the most economically important pest of soybean (*Glycine max* (L.) Merr.) [1]. The annual yield loss caused by SCN is $2 billion [1]. Some agronomic management methods, such as non-host crop rotation and the use of chemical nematicides, may be used to control SCN. The most effective method still is the use of resistant cultivars [2]. However, most commercially available soybean cultivars exhibiting SCN resistance were primarily derived from plant introductions (PIs) ‘PI88788’, ‘PI209332’, ‘PI548402’ and ‘Peking’. These PIs underlie 90% of resistant cultivars [3]. The continual use of so few resistance sources has led to SCN population shifts, resulting in new biotypes [4, 5]. Hence, the selection of a new source of SCN resistance among soybean collections has been challenging.

The inheritance of SCN resistance is complicated [6–14]. The advance of DNA markers has enabled the detection of many quantitative trait loci (QTL) underlying resistance to SCN. To date, several putative QTL have been reported to be associated with resistance to SCN, derived from both cultivated and wild soybeans (*Glycine soja*) [7, 11–14]. These loci have provided resistance to various HG Types (previously races) and have been mapped onto 17 chromosomes (Chr) or linkage groups (LG). Among the identified QTL, *rhg1* alleles a and b on Chr 18 and *Rhg4* allele a on Chr 8 [7] were isolated from ‘PI88788’ and ‘Forrest’, respectively [8, 10]. The copy number variation (CNV) of 31 Kbp DNA segment conferred the SCN resistance of *rhg1* allele b in ‘PI 88788’ and three disparate genes presented on each repeat contribute to SCN resistance [8]. Differentially methylated DNA regions were also identified within *rhg1*, that correlate with soybean cyst nematode resistance [15]. Two point mutations in *Rhg4* of ‘Forrest’ altered a key regulatory property of serine hydroxymethyltransferase. It had been hypothesized that this mutation might result in a nutritional deficiency among female nematodes [10].

SCN HG Type 2.5.7 (race 1) is prevalent in central US and China [16, 17], causing a severe yield loss of soybean. QTLs, associated with the resistance to SCN HG Type 2.5.7, have been identified through linkage mapping using segregating populations. Concibido et al. [18] initially identified QTL with resistance to SCN HG Type 2.5.7 in PI209332 [18]. Among the detected QTL, at least seven loci were identified as adjacent to *rhg1*. To date, most QTL associated with resistance to SCN HG Type 2.5.7 have been detected in North American resistance sources [7, 19], but Chinese resistance sources have been less well studied [1].

Genome-wide association analysis (GWAS), an alternative to linkage analysis, has been widely utilized to analyze the genetic architecture of important traits in crops, such as rice [20], wheat [21], barley [22] and soybean [17, 23]. The development of next-generation sequencing technology and single nucleotide polymorphism (SNP) genotyping technology have greatly promoted the applicability of GWAS [17]. Previously, we identified 19 association signals significantly associated with resistance to two SCN HG Types (HG Type 0 and HG Type 1.2.3.5.7) using 35,760 SNPs [23]. Zhang et al. [17] identified ten SNPs significantly associated with resistance to HG Type 2.5.7 using SoySNP50k iSelect BeadChip assays [17]. However, currently, genome-wide sequencing studies aimed at detecting QTL underlying the resistance to SCN HG Type 2.5.7 are lacking.

The aims of the present study were to identify new sources of HG2.5.7 resistance in 200 diverse soybean collections, primarily collected from China, to obtain insight into the genetic architecture of soybean resistance to SCN HG Type 2.5.7 using 33,194 SNPs and to predict potential candidate genes that might regulate SCN HG Type 2.5.7 resistance in the linked genomic region with peak SNPs.

## Methods

### Genotyping of soybean germplasms

A natural population, including 200 diverse soybean accessions, collected from inside and outside of China, was used for phenotypic evaluation and GWAS. Among the 200 soybean accessions, 179 accessions were selected from 2000 core germplasms, including 88 elite varieties, 35 elite lines and 56 landraces, representing the genetic and geographical diversity of soybean collections in China. The other twenty-one accessions were collected from non-Chinese regions (Additional file 1). These 200 soybean accessions were never tested for resistance to HG Type 2.5.7 before. The genomic DNA of each accession was isolated from the fresh leaves of a single plant according to Wu et al. [24] and partially sequenced using specific locus amplified fragment sequencing (SLAF-seq) methodology [25, 26]. A double enzyme group, comprising *Mse* I (EC 3.1.21.4) and *Hae*III (EC:3.1.21.4) (Thermo Fisher Scientific Inc., Waltham, MA, USA.), was used to digest the soybean genomic DNA into more than 50,000 sequencing tags (approximately 300-500 bp in length). The tags were evenly distributed in unique genomic regions. The sequencing libraries of each accession were constructed based on the sequencing tags. The 45-bp sequence read at both ends of the sequencing tags for each library was obtained using the barcode approach combined with the Illumina Genome Analyzer II (Illumina Inc., San Diego, CA, USA). The Short Oligonucleotide Alignment Program 2 (SOAP2) was used to map raw paired-end reads onto the reference genome (Glycine_ max_Williams_82 8× Release v1.01) [27]. The SLAF groups were obtained after sequencing reads with the same genomic position. Approximately 58,000 high-quality SLAF tags were obtained from each accession. In SNP calling, the MAF threshold was set at 0.04. The genotype was considered heterozygous when the depth of minor allele/the total depth of the sample ≥ 1/3.

### Evaluation of soybean germplasm resistance to SCN HG type 2.5.7

The 200 soybean accessions were used to evaluate the resistance to SCN HG Type 2.5.7 using a previously described inoculation method with minor modifications [23]. The soybean resistance to SCN HG Type 2.5.7 was evaluated in a completely randomized block design with three replications, and five plants in each replication were used, which was repeated twice. Thus, a total of 30 plants for each accession were used for phenotypic analyses. Thirty days after the accessions were inoculated, the cysts and females of the tested accessions were collected and measured. The female index was calculated as FI = (number of cysts and females on detected plant)/(average number of cysts and females on ‘Lee 68’) × 100. FI > 10 and FI < 10 was designated “+” and “-”, respectively [28].

### Population structure evaluation and linkage disequilibrium (LD) analysis

The population structure of the natural soybean population was analyzed using a principal component analysis (PCA) approach in the GAPIT software package [29]. The LD between pairs of SNPs was estimated using squared allele frequency correlations (r^2^) in TASSEL version 3.0 [30]. Only SNPs with a MAF greater than 0.04 and missing data less than 10% were used to estimate LD. In contrast to the GWAS, missing SNP genotypes were not imputed with the major allele prior to LD analysis. Parameters in the program included MAF (≥ 0.04) and the integrity of each SNP (≥ 80%). r-square dropping to half of the maximum value was used to decay measure.

### Association mapping

GLM in TASSEL [30], CMLM and ECMLM in GAPIT [29] were used to conduct GWAS based on 33,194 SNPs from 200 soybean accessions. The *p* value was estimated using the Bonferroni’s method at α ≤ 0.01 (≤ 3.01 × 10^−7^) and 0.05 (≤1.51 × 10^−6^), respectively, and set as the threshold to determine whether a significant association existed [31]. Candidate genes located within the LD block near a SNP peak were identified.

## Results

### Susceptibility of soybean accessions to HG type 2.5.7 infection

**Fig. 1:**
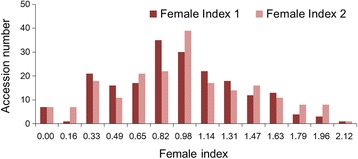
Distribution of the Female Index (FI) among 200 soybean accessions

The female index (FI) value of HG Type 2.5.7 exhibited a continuous distribution in the 200 soybean accessions. A wide range of variation from 0 to 478.7% was observed, with an average FI value of 104.2% (Additional file 1). The phenotypic data showed that sixteen soybean accessions exhibited significant resistance to HG Type 2.5.7. The square root function was used to normalize the phenotypic data of FI value. The kurtosis and skewness was −0.28 and 0.23 for repetition one and −0.48 and 0.28 for repetition two, respectively. The phenotype data of FI value showed near normal distribution after normalization (Fig. 1). The correlation coefficient (r) of FI value of HG Type 2.5.7 between two repeated experiments was quite high, *r* = 0.91 (*P* < 0.01). Thus, the average FI value of two repetitions was used as phenotypic data for GWAS.

### Distribution of markers and linkage disequilibrium

**Fig. 2:**
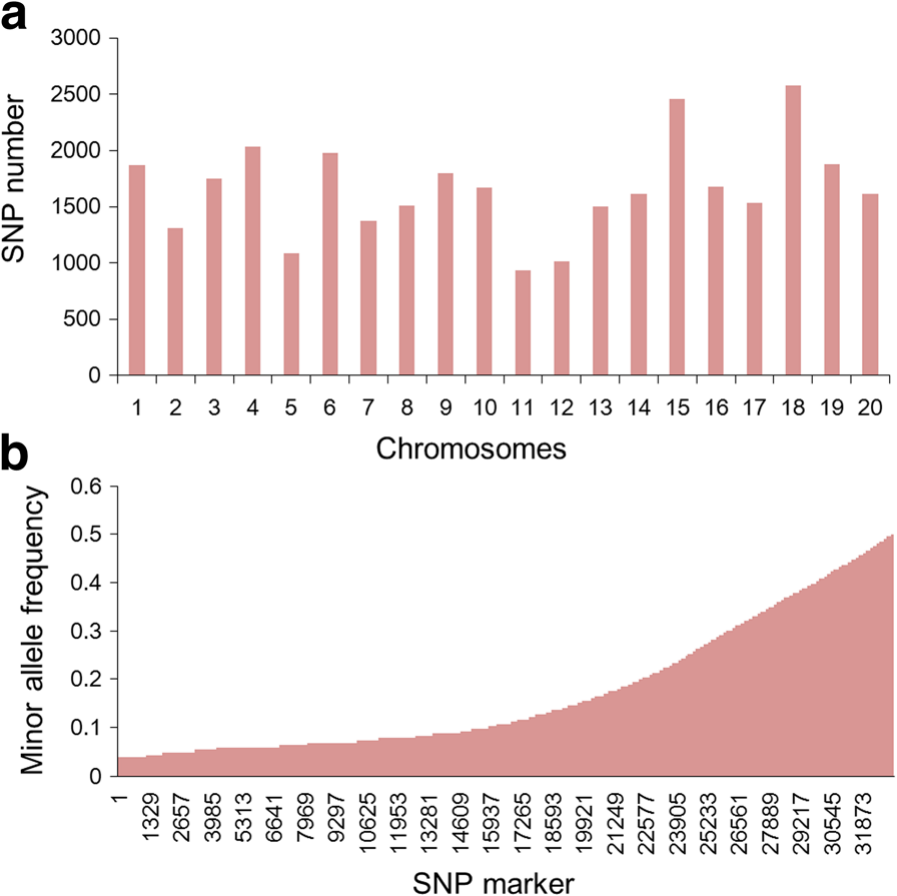
Distribution of the SNP markers across 20 soybean chromosomes (**a**) and minor allele frequency distribution of SNP alleles (**b**)

**Fig. 3:**
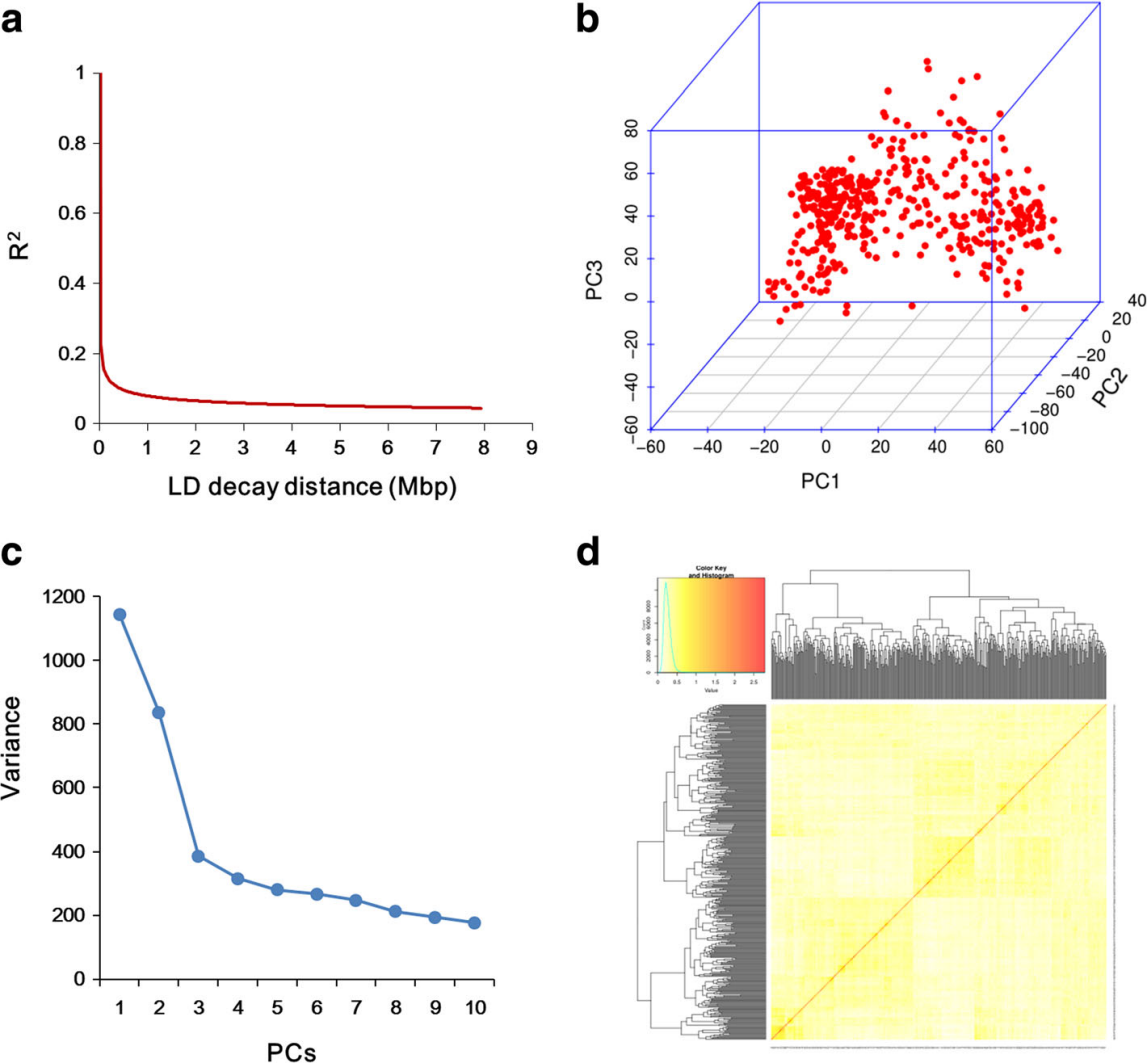
The linkage disequilibrium (LD), principal component and kinship analyses of soybean genetic data. (**a**) The linkage disequilibrium (LD) decay of the genome-wide association study (GWAS) population. (**b**) The first three principal components of the 33,194 SNPs used in the GWAS indicated little population structure among the 200 tested accessions. (**c**) The population structure of the soybean germplasm collection reflected by principal components. (**d**) A heat map of the kinship matrix of the 200 soybean accessions calculated from the same 33,194 SNPs used in the GWAS, suggesting low levels of relatedness among the 200 individuals

A total of 33,194 SNPs, with minor allele frequencies (MAFs) ≥ 0.04, were used to conduct GWAS with a marker density of 28.6 kbp (Fig. 2, Additional file 2). The mean linkage disequilibrium (LD) was 212 kbp (Fig. 3a).

### Quantitative trait nucleotide (QTN) associated with the resistance to HG type 2.5.7 identified by GWAS

**Fig. 4:**
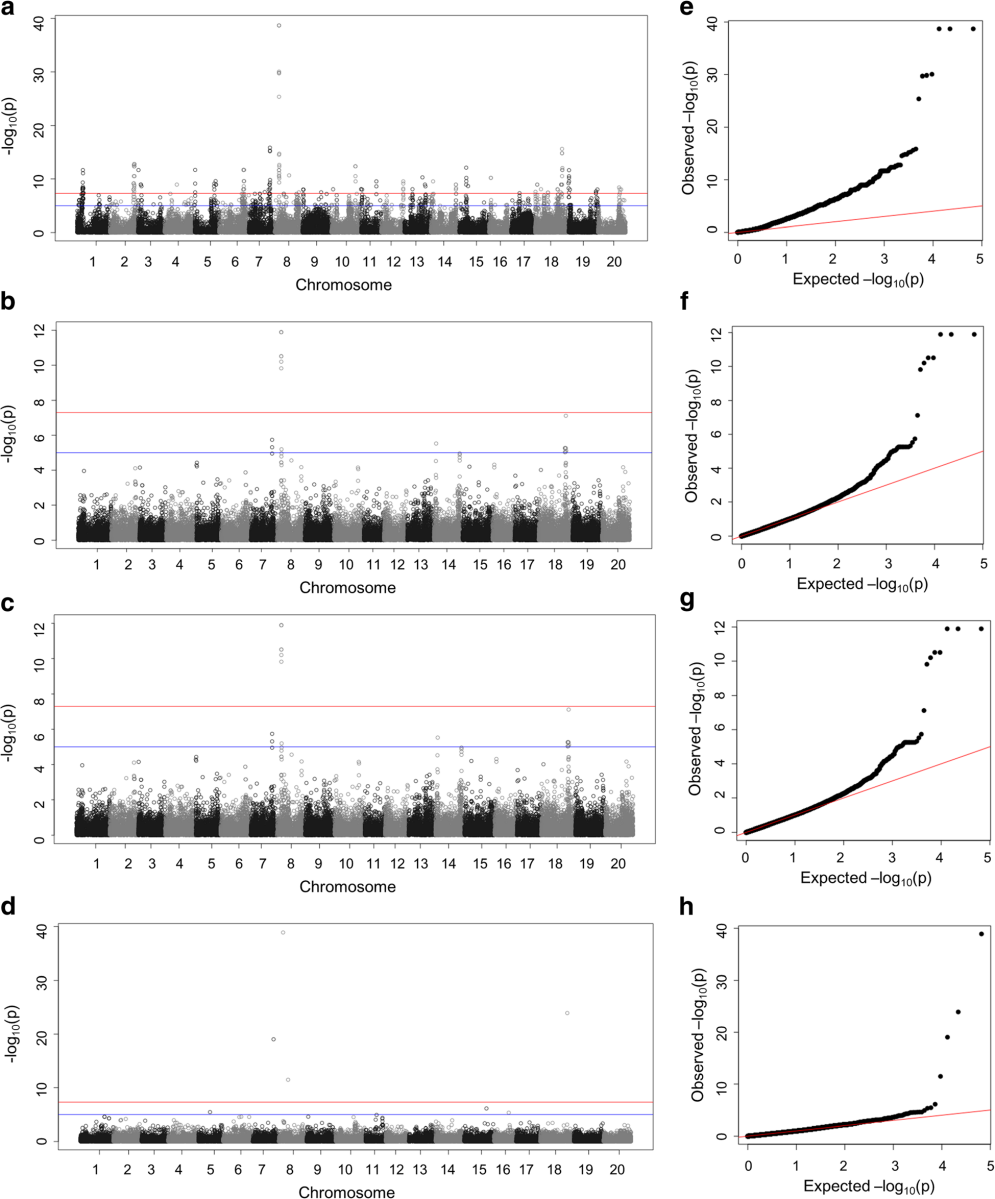
Manhattan and QQ plots of GWAS for soybean susceptibility to HG Type 2.5.7. (**a**-**d**) Negative log10-transformed *P* values of SNPs from a genome-wide scan for soybean resistance to HG Type 2.5.7. were plotted against positions on each of the 20 chromosomes under GLM, CMLM, ECMLM and FarmCPU models. The significant trait-associated SNPs were distinguished by the threshold line and colored in red and blue. (**e**-**h**) QQ plots of GWAS for soybean susceptibility to HG Type 2.5.7. under the above four models

The generalized linear model (GLM) in the JAVA package, the Tassel [30], compressed mixed linear model (CMLM) and the enriched CMLM (ECMLM) model in the R package GAPIT, were utilized in the present study [29]. In addition, a recently developed model selection algorithm [32] was also used. The CMLM and ECMLM considered both population structure and relative kinship [33, 34]. Principal component and kinship analyses were performed using the entire set of SNPs to capture the overall population stratification of the association panel. The first three PCs explained 16.3% of the total genetic variation (Fig. 3b and c). A heatmap of the kinship matrix with genetic relatedness among the soybean accessions calculated from 33,194 SNPs used in the GWAS suggested low levels of relatedness among the 200 individuals (Fig. 3d).The quantile-quantile (QQ) plot showed that the observed *p* values seriously deviated from the expected *p* values for the GWAS result based on GLM method compared with that of the CMLM, ECMLM and FARMCPU methods (Fig. 4e-h). Since the observed and expected *P*-values differed substantially only for a few SNPs, the QQ plot supported the CMLM, ECMLM and FARMCPU as the appropriate GWAS models. Only involved population structures and kinship (CMLM, ECMLM and FARMCPU) showed significant control of the influence on the results of GWAS resulting from population structure and kinship. Therefore, the GWAS results using compressed MLM and FARMCPU methods were emphatically investigated.

Through GWAS, a total of thirteen SNPs were found to be associated with resistance to HG Type 2.5.7. Of them, ten SNPs were simultaneously detected using CMLM and ECMLM. Another three SNPs that located on Chr7, Chr 8 and Chr15 were detected under FARMCPU method (Table 1). The association signals were distributed on five chromosomes, including Chr7, Chr8, Chr14, Chr15 and Chr18. Among the ten significantly associated SNPs from CMLM and ECMLM, seven SNPs were located on Chr8. The other three SNPs were located on Chr18, Chr7 and Chr14, respectively. Four of the detected association signals were novel loci that were firstly found by the present study and another nine association signals overlapped with the known QTL underlying resistance to SCN (Table 2). Two stable loci, rs7671170 on Chr8 and rs46625879 on Chr18, were simultaneously identified using the three models. The effect of beneficial allele of each peak SNP associated with SCN resistance was analyzed. The result indicated that the average FI values of accessions with resistant alleles were significantly lower than that of the accessions with susceptible alleles for the all thirteen association signals. They were also lower than the average FI value of the whole association panel (Table 1). Therefore, these resistant alleles could be useful for marker-assistant selection (MAS) of SCN resistance and these loci could be valuable for the isolation of candidate genes underlying the resistance to HG Type 2.5.7.

**Table 1:** Peak SNP and benefical allele associated with resistance to the Hg Type 2.5.7 (race 1) identified by GWAS

SNP	Chromosome	Position	-log_10_(p)	MAF	Model	Resistant allele	Susceptible alleles	Average FI of accessions with resistant allele	Average FI of accessions with susceptible allele	Average FI of population
rs7631207	8	7,631,207	11.89	0.12	CMLM	C	G	22.71	111.07	104.19
rs7640250	8	7,640,250	11.89	0.12	CMLM	C	G	22.71	111.07	104.19
rs7671170	8	7,671,170	11.89	0.12	CMLM	G	C	22.71	111.07	104.19
rs7662003	8	7,662,003	10.51	0.12	CMLM	T	C	39.36	110.13	104.19
rs7664479	8	7,664,479	10.51	0.14	CMLM	C	A	39.02	109.75	104.19
rs7622492	8	7,622,492	10.2	0.11	CMLM	A	G	24.19	110.39	104.19
rs7661660	8	7,661,660	9.82	0.12	CMLM	C	A	39.36	109.97	104.19
rs46625879	18	46,625,879	7.12	0.1	CMLM	G	T	44.55	107.59	104.19
rs36423980	7	36,423,980	5.74	0.17	CMLM	A	G	49.27	112.53	104.19
rs3853672	14	3,853,672	5.53	0.1	CMLM	A	T	53.89	107.40	104.19
rs7631207	8	7,631,207	11.89	0.12	ECMLM	C	G	22.71	111.07	104.19
rs7640250	8	7,640,250	11.89	0.12	ECMLM	C	G	22.71	111.07	104.19
rs7671170	8	7,671,170	11.89	0.12	ECMLM	G	C	22.71	111.07	104.19
rs7662003	8	7,662,003	10.51	0.12	ECMLM	T	C	39.36	110.13	104.19
rs7664479	8	7,664,479	10.51	0.14	ECMLM	C	A	39.02	109.75	104.19
rs7622492	8	7,622,492	10.2	0.11	ECMLM	A	G	24.19	110.39	104.19
rs7661660	8	7,661,660	9.82	0.12	ECMLM	C	A	39.36	109.97	104.19
rs46625879	18	46,625,879	7.12	0.1	ECMLM	G	T	44.55	107.59	104.19
rs36423980	7	36,423,980	5.74	0.17	ECMLM	A	G	49.27	112.53	104.19
rs3853672	14	3,853,672	5.53	0.1	ECMLM	A	T	53.89	107.40	104.19
rs7671170	8	7,671,170	38.91	0.12	FARMCPU	G	C	22.71	111.07	104.19
rs46625879	18	46,625,879	23.93	0.1	FARMCPU	G	T	44.55	107.59	104.19
rs35898587	7	35,898,587	19.04	0.07	FARMCPU	A	C	48.30	107.38	104.19
rs16268025	8	16,268,025	11.48	0.06	FARMCPU	A	G	71.46	106.50	104.19
rs38522986	15	38,522,986	6.12	0.05	FARMCPU	A	G	52.14	107.16	104.19

**Table 2:** Significant SNPs and predicted candidate genes associated with SCN HG Type 2.5.7 resistance in soybean

SNP	Chr	Position	Model	QTLs	Gene	Distance to SNP (Kbp)	Functional description	Expression pattern
rs35898587	7	35,898,587	FARMCPU	-	Glyma.07G190900	46.68	sphingosine kinase 1	
					Glyma.07G191000	42.52	Vacuolar protein sorting-associated protein VPS28 family protein	
					Glyma.07G191100	33.66	endonuclease 4	
					Glyma.07G191200	29.08	alternative NAD(P)H dehydrogenase 2	
					Glyma.07G191500	2.12	HAL2-like	
rs36423980	7	36,423,980	CMLM, ECMLM	-	Glyma.07G195400	28.22	RING/U-box superfamily protein	
					Glyma.07G195500	12.78	transcription factor-related	
					Glyma.07G196000	28.36	RING membrane-anchor 1	
					Glyma.07G193900	174.20	Dof-type zinc finger DNA-binding family protein	regulated by SCN
					Glyma.07G196500	59.39	phosphate 2	regulated by SCN
					Glyma.07G196800	85.03	lipoxygenase 3	regulated by SCN
rs7631207	8	7,631,207	CMLM, ECMLM	Mattews et al. 1998 [40]; Yuan et al. 2002 [42]; Guo et al. 2006 [9]; Vuong et al. 2011 [41]	Glyma.08G099400	24.52	CBL-interacting protein kinase 23	
rs7640250	8	7,640,250	CMLM, ECMLM		Glyma.08G099700	32.06	Metallo-hydrolase/oxidoreductase superfamily protein	
rs7671170	8	7,671,170	CMLM, ECMLM, FARMCPU		Glyma.08G100100	2.50	auxin response factor 8	
					Glyma.08G100700	54.67	RING/U-box superfamily protein	
					Glyma.08G100800	60.21	Leucine-rich repeat protein kinase family protein	
rs7662003	8	7,662,003	CMLM, ECMLM					
rs7664479	8	7,664,479	CMLM, ECMLM					
rs7622492	8	7,622,492	CMLM, ECMLM		Glyma.08G097300	184.48	Aldolase-type TIM barrel family protein	regulated by SCN
rs7661660	8	7,661,660	CMLM, ECMLM					
rs16268025	8	16,268,025	FARMCPU	-	Glyma.08G200800	21.21	protein kinase family protein / peptidoglycan-binding LysM domain-containing protein	
					Glyma.08G201000	9.76	hydroxyproline-rich glycoprotein family protein	
					Glyma.08G201100	1.89	HPT phosphotransmitter 4	
					Glyma.08G200100	84.82	HAD superfamily, subfamily IIIB acid phosphatase	regulated by SCN
					Glyma.08G200200	80.21	HAD superfamily, subfamily IIIB acid phosphatase	regulated both constitutively and by SCN
					Glyma.08G202300	57.12	Integrase-type DNA-binding superfamily protein	regulated by SCN
rs3853672	14	3,853,672	CMLM, ECMLM	-	Glyma.14G048600	74.83	disease resistance protein (TIR-NBS-LRR class), putative	
					Glyma.14G049500	15.54	ethylene-forming enzyme	
					Glyma.14G047900	179.55	Leucine-rich repeat receptor-like protein kinase family protein	regulated by SCN
					Glyma.14G051600	196.06	Copper transport protein family	regulated by SCN
rs38522986	15	38,522,986	FARMCPU	Kabelka et al. 2005 [43]				
rs46625879	18	46,625,879	CMLM, ECMLM, FARMCPU	Winter et al. 2007 [12]	Glyma.18G193200	65.89	laccase 7	
					Glyma.18G193300	49.11	laccase 8	
					Glyma.18G193400	3.01	Laccase/Diphenol oxidase family protein	

Note: the expression pattern was according to Wan et al. BMC Genomics [39]

### Prediction of candidate genes for SCN resistance to HG type 2.5.7

The candidate genes inferred to underlie resistance to HG Type 2.5.7 were evaluated. Genes located in the 200 kbp genomic region of each peak SNP in the reference soybean genome (version a2.v1 of Williams 82, www. phetozome.net) were considered as candidate genes according to the average LD decay distance of 212 kbp for the GWAS panel.

**Fig. 5:**
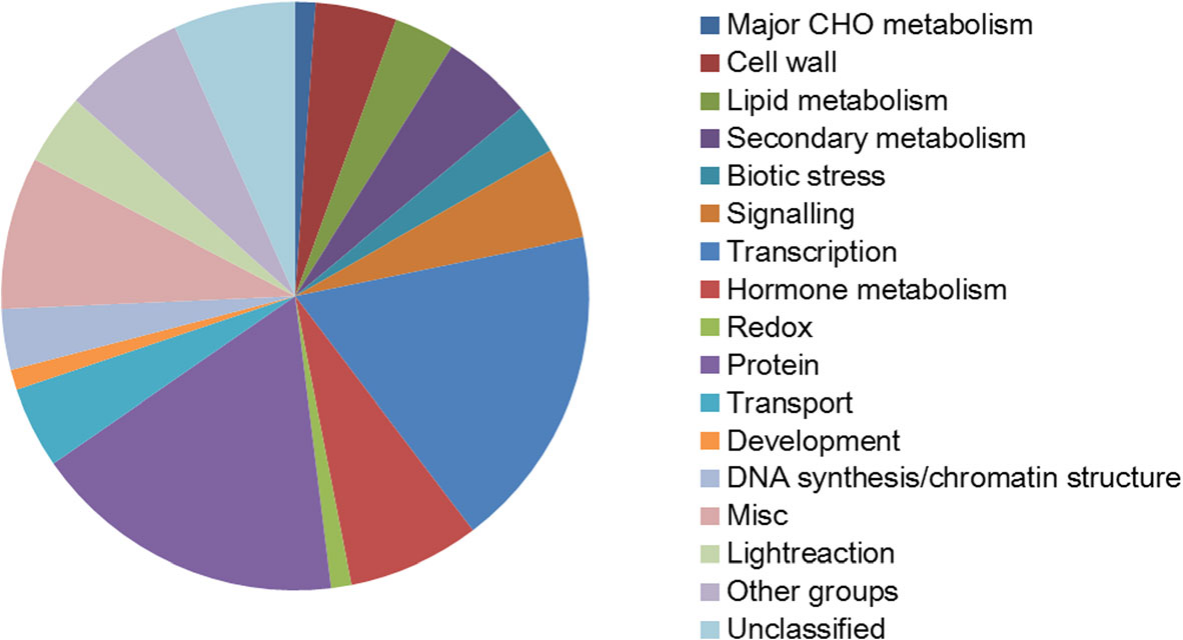
Functional categories of the predicted resistant genes to HG Type 2.5.7

A total of 248 soybean genes were identified in the flanking region of each peak SNP (Additional file 3). Among these genes, 53 genes had no functional annotation, and seven genes belonged to the domains of unknown function families. To predict potential functions of genes in the flanking region of SNPs associated with the resistance to SCN, the 196 genes were grouped into the following functional categories using MapMap [35]: cell wall metabolism (eight genes), lipid metabolism (six genes), secondary metabolism (nine genes), biotic stress (five genes), signaling (nine genes), transcription regulation (transcription factors, TFs, 32 genes), hormonal metabolism (13 genes), redox group (two genes), protein modification and degradation (31 genes), transport (eight genes), development (two genes), DNA synthesis or chromatin structure (six genes), miscellaneous group (15 genes: 3 cytochrome P450 genes, 2 GDSL-motif lipase, 2 nitrile lyases etc.), light reaction (seven genes), other groups of genes (12 genes), and unclassified genes (12 genes) (Fig. 5). Of them, many genes have been implicated in plant disease defense or plant disease resistance pathways, including protein kinase family (belonging to the signal group), leucine-rich repeat-containing proteins and receptor-like protein. Some domain types, such as cytochrome P450s (belonging to the miscellaneous group), zinc fingers and RING (belonging to the transcription factors), have been implicated in soybean responses to SCN [23]*.* To more accurately predict the candidate genes, the genes in a 50 kbp genomic region of each side of the peak SNP were further selectively analyzed. A total of 21 candidate genes were eventually verified. Glyma.07G195500, encoding a transcription factor-related gene, was 12.78 kbp away from SNP rs36423980 on Chr7. Glyma.07G195400 and Glyma.07G196000, with RING domains, were associated with SCN resistance. Gruenwald et al. reported that the auxin-inducible transcription factor AtWRKY23 was expressed during the infection of Arabidopsis roots with *H. schachtii* and demonstrated that the regulation of AtWRKY23 was controlled through auxin response factor 7 (ARF7) and the ARF19 pathway [36]. Herein, an auxin response factor gene (Glyma.08G100100) 2.4 kbp from rs7671170 on Chr 8 was implicated in the soybean reaction to SCN infection. In the present study, Laccase genes (Glyma.18G193200, Glyma.18G193300, and Glyma.18G193400), which participate in lignin synthesis and phenolic compound metabolism in plants [37], might also contribute to HG Type 2.5.7 resistance in soybean. Ithal et al. tested three time points after SCN feeding (2, 5, and 10 dpi) and also noted an increase in the expression of genes involved in lignin biosynthesis and phenolic compound metabolism [38]. Except for the above genes, nine genes out of the 196 were regulated by SCN in different soybean lines according to the report by Wan et al. [39]. Of them, Leucine-rich repeat receptor-like protein kinase family protein (Glyma.14G047900) might be involved in SCN resistance since the gene showed up-regulated after SCN inoculation (Table 2) [39].

## Discussion

SCN is estimated to cause the greatest yield loss in soybean compared with other pests worldwide [7]. Genes conferring durable resistance to SCN might exist in the soybean germplasms of China, where soybean originated [23]. In the present study, a total of 200 soybean accessions primarily collected inside China were tested. Among these accessions, 16 accessions showed high resistance to SCN HG Type 2.5.7, most of which were landraces with specific elite agronomic traits. Therefore, these resistance sources have great potential value for future breeding for SCN resistance.

To date, numbers of SCN-resistant QTL have been reported [7]. Most of which were verified using different cross populations from limited resistance sources. Two major QTL across multiple resistant sources were *rhg1* and *Rhg4* [7]. Additionally, QTL qSCN11, located on Chr11, has also been consistently identified from PI 437654, PI 90763 and PI 404198B [2]. In the present study, a total of thirteen SNPs distributed on five chromosomes (Chr 7, 8, 14, 15 and 18) were associated with SCN HG Type 2.5.7 resistance. Among the thirteen association signals, nine SNPs overlapped with or were located near known QTL (Table 2). A resistant genomic region in Chr8, containing the gene loci of rs7631207, rs7640250, rs7671170, rs7662003, rs7664479, rs7622492 and rs7661660, were significantly associated with SCN resistance to HG Type 2.5.7, and the relation between these genomic regions and SCN resistance has been reported in previous studies [9, 40–42]. Similarly, two SNPs, rs38522986 and rs46625879, located on Chr15 and Chr18, respectively, were identified inside two marker intervals previously reported by Kabelka et al. [43] and Winter et al. [12]. Moreover, two genomic regions (rs7671170 on Chr8 and rs46625879 on Chr18) could stably be identified using three models, including CMLM, ECMLM and FARMCPU, which further verified the importance of these two genomic regions for resistance to HG Type 2.5.7. These consistent genomic regions in the present and previous studies showed that Chr8, Chr15 and Chr18 might play important roles in conferring SCN resistance in the soybean germplasms of China and North America.

Major QTL, *rhg1* and *Rhg4*, were valuable resources for SCN resistance, but were frequently not durable, reflecting shifts in the SCN population that resulted in a loss of SCN resistance in major QTL [2]. Breeding soybean with durable resistance to SCN through the identification and utilization of novel QTL is an effective strategy to cope with the loss of SCN resistance [2]. One novel QTL on Chr10 (qSCN10) was identified in PI 567516C [4], which could confer high SCN resistance to soybean lacking the two known major genes, *rhg1* and *Rhg4*. Additionally, four novel QTL (rs35898587 and rs36423980 on Chr7, rs16268025 on Chr8, and rs3853672 on Chr14) were also identified, which were significantly different from the major QTL reported in previous studies. Although the molecular mechanisms of the novel resistance loci were not clear, these loci possessed high potential to breed cultivars with durable resistance to SCN through the pyramid of the novel and previously reported QTL [7].

Presently, for the molecular mechanism of SCN resistance genes, only two genes, *rhg1* and *Rhg4,* were clearly associated with the molecular mechanism of SCN resistance [8, 10], and other candidate genes or QTL underlying SCN resistance were less investigated. Thus, it was difficult to predict and confirm the SCN candidates from large QTL intervals with a number of genes. However, GWAS could still offer some valuable clues to identify and confirm SCN resistance genes, particularly within a linkage disequilibrium (LD) block (150-200 kbp in length on average) [23, 44]. In the present study, a total of 196 potential candidate genes, located in 200 kbp flanking regions up- and downstream of thirteen peak SNPs, possessed the canonical SCN resistance domains (including cytochrome P450s, zinc fingers and RING) [23], which are involved in plant disease responses or plant disease resistance pathways. Among these candidate genes, six genes (Glyma.07G195400, Glyma.07G196000, Glyma.08G100100, Glyma.18G193200, Glyma.18G193300 and Glyma.18G193400) have been reported to be responsible for SCN resistance [23]. Furthermore, 15 novel genes (Glyma.07G190900, Glyma.07G191000, Glyma.07G191100, Glyma.07G191200, Glyma.07G191500, Glyma.07G195500, Glyma.08G099400, Glyma.08G099700, Glyma.08G100700, Glyma.08G100800, Glyma.08G200800, Glyma.08G201000, Glyma.08G201100, Glyma.14G048600, and Glyma.14G049500), located in 50 kbp flanking regions up- and downstream of peak SNPs, were associated with SCN HG Type 2.5.7 resistance in the present study. The clear function of these candidates should be discussed in future studies.

## Conclusions

A total of sixteen novel soybean accessions were identified with significant resistance to HG Type 2.5.7. The multiple beneficial alleles and candidate genes from novel resistant germplasms might be valuable for the breeding of cultivars with long-lasting resistance to SCN.

## Additional files

Additional file 1: Source and phenotype data of 200 soybean accessions. (XLSX 19 kb)

Additional file 2: SNP list of 200 soybean accessions. (7Z 1043 kb)

Additional file 3: Gene models in the flanking regions of peak SNP. (XLSX 26 kb)

## Abbreviations

CMLM: Compressed mixed linear model
ECMLM: enriched compressed mixed linear model
GLM: General linear model
GWAS: Genome-wide association study
LD: Linkage disequilibrium
MAF: Minor allele frequency
PCA: principal component analysis
QTL: Quantitative Trait Locus.
SCN: Soybean cyst nematode
SLAF-seq: Specific Locus Amplified Fragment Sequencing
SNPs: Single nucleotide polymorphisms
